# Interspecific Variation in Compensatory Regrowth to Herbivory Associated with Soil Nutrients in Three *Ficus* (Moraceae) Saplings

**DOI:** 10.1371/journal.pone.0045092

**Published:** 2012-09-12

**Authors:** Jin Zhao, Jin Chen

**Affiliations:** 1 Key Laboratory of Tropical Forest Ecology, Xishuangbanna Tropical Botanical Garden, Chinese Academy of Sciences, Mengla, Yunnan, China; 2 Graduate School of the Chinese Academy of Sciences, Beijing, China; Centro de Investigación y de Estudios Avanzados, Mexico

## Abstract

Plant compensatory regrowth is an induced process that enhances plant tolerance to herbivory. Plant behavior against herbivores differs between species and depends on resource availability, thus making general predictions related to plant compensatory regrowth difficult. To understand how soil nutrients determine the degree of compensatory regrowth for different plant species, we selected saplings of three *Ficus* species and treated with herbivore insects and artificial injury in both glasshouse conditions and in the field at two soil nutrient levels. Compensatory regrowth was calculated by biomass, relative growth rate and photosynthetic characteristics. A similar pattern was found in both the glasshouse and in the field for species *F. hispida*, where overcompensatory regrowth was triggered only under fertile conditions, and full compensatory regrowth occurred under infertile conditions. For *F. auriculata*, overcompensatory regrowth was stimulated only under infertile conditions and full compensatory regrowth occurred under fertile conditions. *Ficus racemosa* displayed full compensatory regrowth in both soil nutrient levels, but without overcompensatory regrowth following any of the treatments. The three *Ficus* species differed in biomass allocation following herbivore damage and artificial injury. The root/shoot ratio of *F. hispida* decreased largely following herbivore damage and artificial injury, while the root/shoot ratio for *F. auriculata* increased against damage treatments. The increase of shoot and root size for *F. hispida* and *F. auriculata*, respectively, appeared to be caused by a significant increase in photosynthesis. The results indicated that shifts in biomass allocation and increased photosynthesis are two of the mechanisms underlying compensatory regrowth. Contrasting patterns among the three *Ficus* species suggest that further theoretical and empirical work is necessary to better understand the complexity of the plant responses to herbivore damage.

## Introduction

Growing evidence suggests that compensatory regrowth is a common tolerance strategy in plants in response to herbivore damage [1]–[4]. Compensatory regrowth is often achieved by mobilizing resource allocation or physiological function to reduce the impacts of damage on fitness relative to undamaged plants [1], [2], [5]–[7]. Following herbivore feeding, biomass of damaged plants could be larger (overcompensatory regrowth), equal (full compensatory regrowth) and less than (under compensatory regrowth) undamaged plants [2]. Differing from the constitutive resistance that plants invest in prior to herbivory damage, compensatory regrowth is an induced process following herbivore attack. It has been widely acknowledged that compensatory regrowth depends on resource availability in the plant's environment [1], [8], [9].

Compensatory regrowth is a heritable trait and varies markedly among plant species [2], [10], [11]. Early theories assumed that compensatory regrowth mostly occurred in herbs because of the faster growth rate compared to woody plants, but later studies demonstrated that many woody plants are also able to compensate or even overcompensate for biomass losses caused by herbivores [10], [12]–[15]. Plants along a successional gradient may also differ in their herbivore defense strategy and degree of tolerance [16]–[18]. Early to middle successional plants often experience higher levels of herbivory [19] and early successional plants tend to show rapid leaf turnover, comparatively little investment in defensive secondary compounds, and rapid regrowth when compensating for tissue loss [20]. In contrast, late successional plant species have intrinsically slower growth rates [21], [22] and possibly lower levels of compensatory regrowth.

Many other factors may also determine the degree of compensatory regrowth such as type, frequency and severity of damage, and the availability of nutrients [8], [20], [21]. Previous studies have suggested that plant defense may be responsive to insect feeding but not to physical damage alone [22], [23]. For example, a study on *Nicotiana sylvestris* (Solanaceae) indicated that higher concentrations of jasmonic acid (JA), which is known to mediate wound responses in plants, resulted mainly from herbivory by *Manduca sexta* (L.) larvae than by mechanical damage [24]. For a better understanding of induced defense by plants, it is therefore necessary to distinguish whether the induced response is stimulated by herbivores, mechanical injuries or both. A plant's compensatory regrowth against herbivore damage also depends on surrounding resource availability. Some studies have shown that greater compensatory regrowth occurred in high-resource environments [2], [25]–[30], while other studies displayed contradictory results in which plants showed greater compensatory regrowth in relatively stressful environments [29], [31]–[33]. Until now, many attempts for a general explanation of plant compensatory regrowth under different environments have been proposed [8], [9], [20], [34]; however, it appears difficult to make general predictions on compensatory regrowth following herbivore damage. For this reason, interspecific comparisons can help elucidate how plant species may evolve higher levels of compensatory regrowth [2].

We were particularly interested in examining how potential mechanisms of compensatory regrowth for woody plants are influenced by plant species and resource availability, since the majority of research in this field has focused on herbs and the conclusions may not pertain to woody species [7], [35]. Woody species in general have a proportionately large capacity for storage of carbon and nutrient reserves compared with herbaceous species [36]. The allocation and accumulation of these reserves within the tree following defoliation is of particular interest because it may provide insights into why defoliation sometimes has little or no effect on growth [37], [38]. In this study, we report the effect of resource availability on compensatory regrowth and the potential mechanisms of compensatory regrowth against herbivore damage in *Ficus* saplings.

*Ficus* (Moraceae) is one of the largest genera of woody plants in the tropics and shows diverse adaptations to different habitats [27]. Three *Ficus* species that are commonly distributed along rainforest edges or beside roads were studied: *F. hispida*, *F. racemosa* and *F. auriculata*. Previous findings suggest that these *Ficus* species receive significantly different levels of damage from herbivorous insects both in the field and in glasshouse experiments [39], and show interspecific variation in morphological and chemical defense [39], [40]. In a common garden experiment, *F. hispida* suffered significantly more severe herbivore damage than the other two species [39]. And, the typical pioneer species *F. hispida* has considerably more pubescence on both the upper and lower leaves and lower C/N than the intermediate successional species *F. auriculata* [39]. Additionally, the amount of volatiles released by the three *Ficus* species were significantly different following herbivore damage [40].

To understand how soil nutrients determine the degree of compensatory regrowth of these *Ficus* species following herbivore damage and the potential mechanisms of compensatory regrowth, one field and one glasshouse experiment in which saplings of each species were treated with herbivores and artificial injury at two different soil nutrient levels were established. We predicted that: 1) compensatory regrowth of the three *Ficus* species would be greater under fertile conditions than infertile conditions; 2) different species may display different patterns in compensatory regrowth associated with resource availability; 3) herbivore damage causes different patterns in compensatory regrowth compared with artificial injury. Broadly, we wish to examine whether the three *Ficus* species show a general pattern in compensatory regrowth against herbivores under different levels of resource availability.

## Results

### Compensatory regrowth

**Figure 1:**
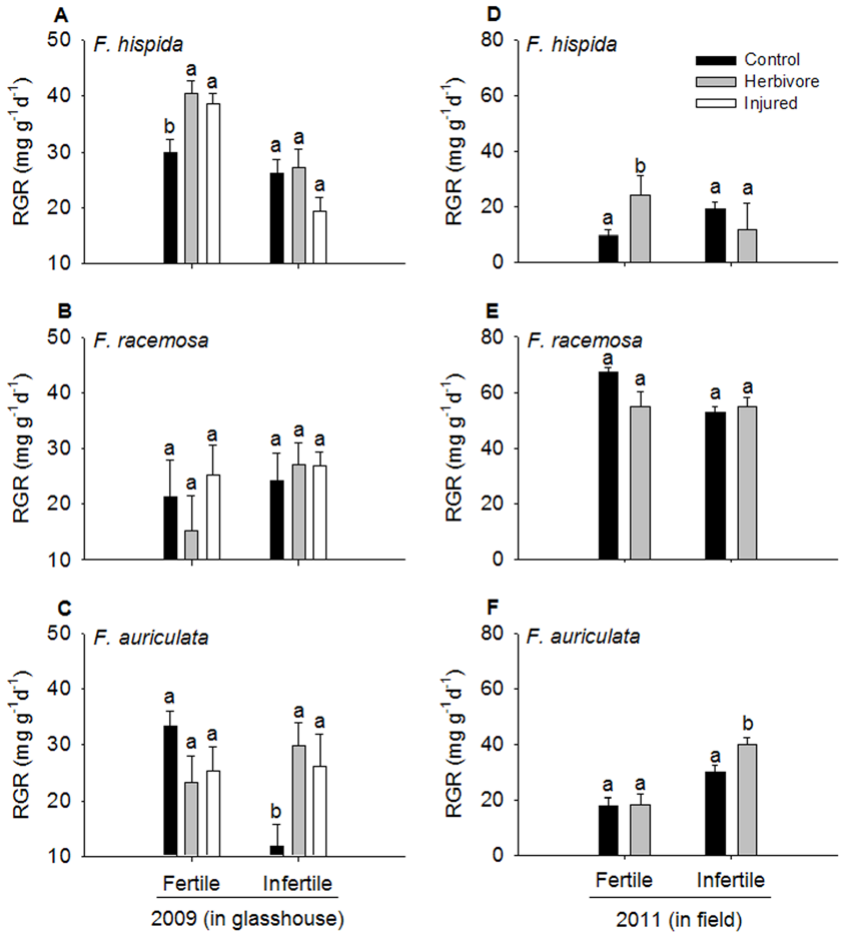
Effect of treatments on RGR of *Ficus* saplings under two soil nutrient levels. Significant differences between treatments are marked with different letters (mean ± SE, n = 5 in 2009, n = 12 in 2011, *P*<0.05). RGR, relative growth rate (mg·g^−1^·d^−1^).

Relative growth rate (RGR) differed significantly among species (F = 3.63, *P* = 0.009 in 2009; F = 64.52, *P*<0.001 in 2011), and RGRs of saplings under fertile condition were higher significantly than these in infertile condition (F = 21.95, *P*<0.001 in 2009; F = 13.26, *P*<0.001 in 2011). But damage treatments did not affect RGRs siginficantly (F = 1.06, *P* = 0.15 in 2009; F = 1.72, *P* = 0.19 in 2011). RGRs were also significantly affected by the interaction between species and soil nutrient level (F = 4.56, *P*<0.001 in 2009; F = 7.07, *P*<0.001 in 2011), and the interaction among species, soil nutrient level and treatments (F = 1.89, *P* = 0.022 in 2009; F = 6.07, *P* = 0.003 in 2011). Twenty days after damage treatments were carried out, RGR of *F. hispida* and *F. auriculata* saplings under infertile conditions were lower than those under fertile conditions regardless of the herbivore treatment, but not for *F. racemosa* (Fig. 1). Under fertile conditions, RGR of *F. hispida* increased significantly after herbivore damage and artificial injury (Fig. 1A, D), while for *F. racemosa* and *F. auriculata*, RGR did not differ significantly among the three treatments (Fig. 1B, C, E, F). Of the saplings planted in the infertile soil, only *F. auriculata* showed a significant increase in RGR after herbivore damage and artificial injury (Fig. 1C, F).

**Figure 2:**
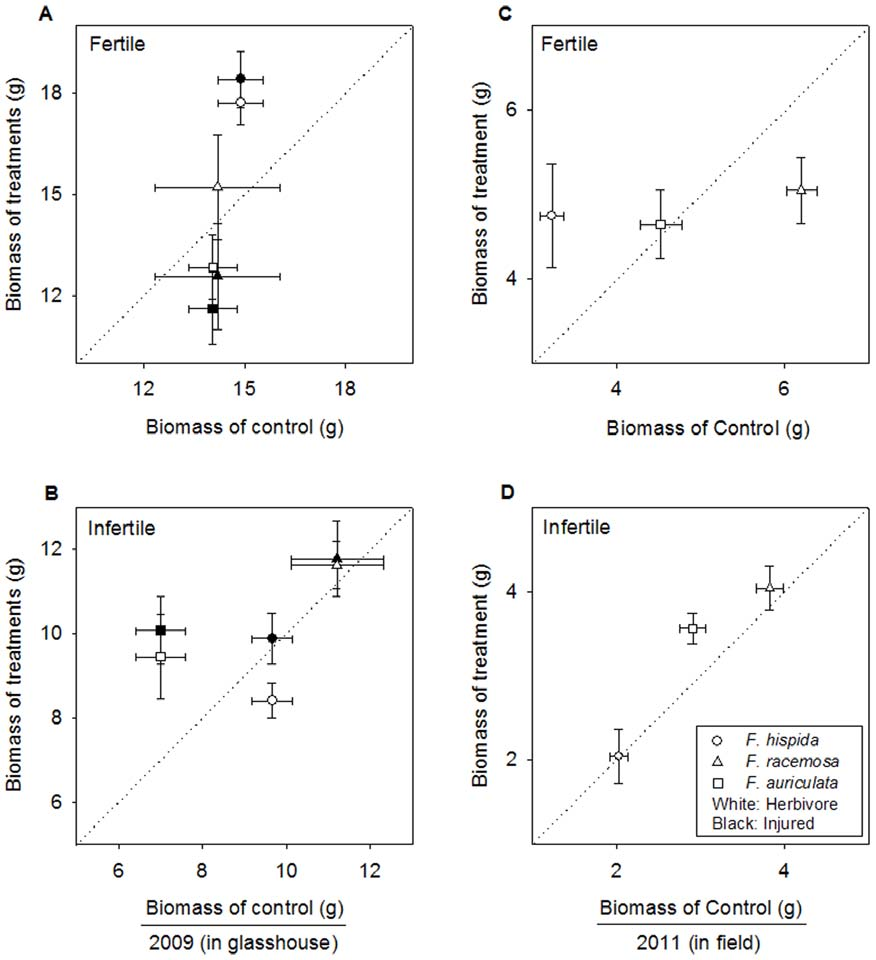
Compensatory growth of three *Ficus* species in response to herbivore treatment. Plant biomass above the line indicates overcompensatory regrowth, on the line indicates fullcompensatory regrowth, below the line indicates undercompensatory regrowth (Biomass in the damaged state  =  biomass in control state, slope  = 1) (n = 5 in 2009, n = 12 in 2011).

*Ficus hispida* showed overcompensatory regrowth after herbivore damage and artificial injury under fertile soil conditions, but showed full compensatory regrowth under infertile conditions (Fig. 2). *F. racemosa* displayed full compensatory regrowth after herbivore damage and artificial injury under both soil nutrient levels. In contrast, *F. auriculata* displayed overcompensatory regrowth only in infertile soil after herbivore damage and artificial injury but full compensatory regrowth under fertile conditions.

By comparing the total biomass of undamaged saplings in glasshouse experiment, we also found that *F. hispida* and *F. auriculata* saplings were significantly negatively affected by soil nutrient level (*F. hispida*: 14.89±0.67 g under fertile and 9.66±0.49 g under infertile conditions (n = 5, *P*<0.0001); *F. auriculata*: 14.06±0.72 g under fertile and 7.01±0.60 g under infertile conditions (n = 5, *P*<0.0001)). However, the total biomass of undamaged saplings of *F. racemosa* was similar between the two soil nutrient levels (14.21±1.86 g under fertile and 11.21±1.10 g under infertile conditions (n = 5, *P* = 0.20).

### Biomass allocation

**Figure 3:**
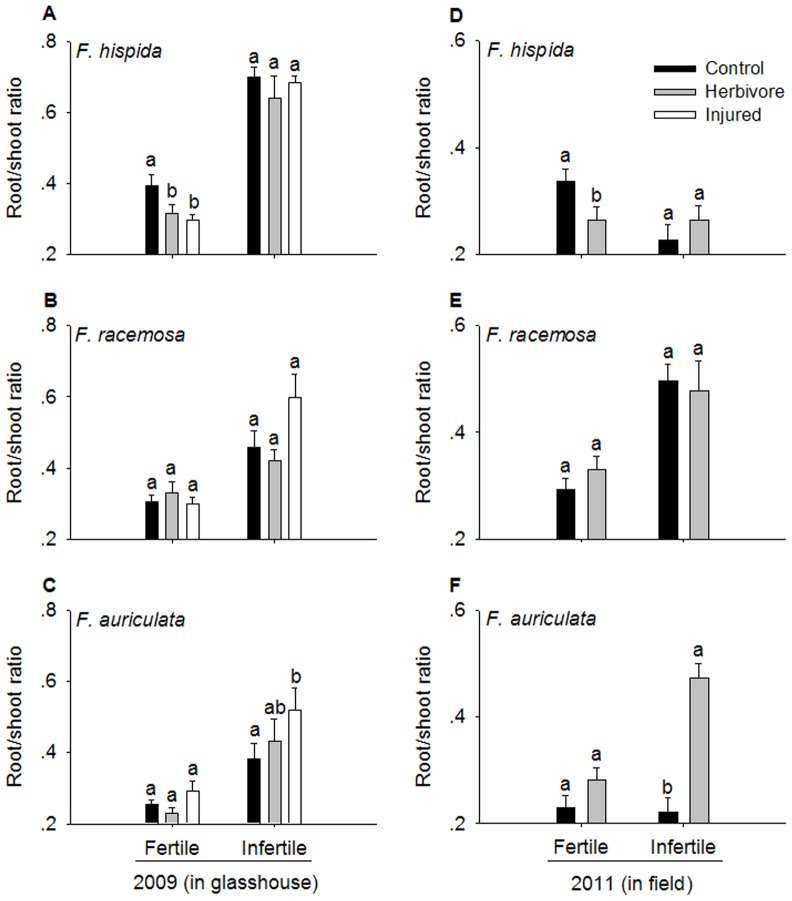
Effect of treatments on root/shoot ratio in *Ficus* saplings under two soil nutrient levels. Significant differences between treatments are marked with different letters (mean ± SE, n = 5 in 2009, n = 12 in 2011, *P*<0.05).

In the glasshouse experiment, root/shoot ratio was affected significantly by species (F = 33.62, *P*<0.001), soil nutrient level (F = 146.54, *P*<0.001), damage treatments (F = 3.38, *P* = 0.04), and interaction between species and nutrient level (F = 3.95, *P* = 0.024). In the field, species (F = 20.71, *P*<0.001), soil nutrient level (F = 17.71, *P*<0.001), treatments (F = 8.22, *P* = 0.005) and their interactions (F = 4.95, *P* = 0.008) had a significant effect on the root/shoot ratio. Root/shoot ratio of *F. hispida* saplings decreased remarkably following herbivore and artificial injury treatments in fertile soil (Fig. 3A, D). However, root/shoot ratio of *F. racemosa* did not change considerably after herbivore feeding or artificial injury under either soil nutrient level (Fig. 3B, E). Only *F. auriculata* saplings showed a prominent increase in root/shoot ratio after herbivore and artificial damage in infertile soils (Fig. 3C, F).

### Natural herbivore feeding in the field

In the field experiment, the consumed area of the whole sample of leaves was not related to the *Ficus* species, soil nutrient or their interaction (12.38±3.25% for *F. hispida*, 5.88±1.16% for *F. racemosa*, 6.50±1.28% for *F. auriculata*; n = 4).

### Biomass and Photosynthetic gas exchange

**Figure 4:**
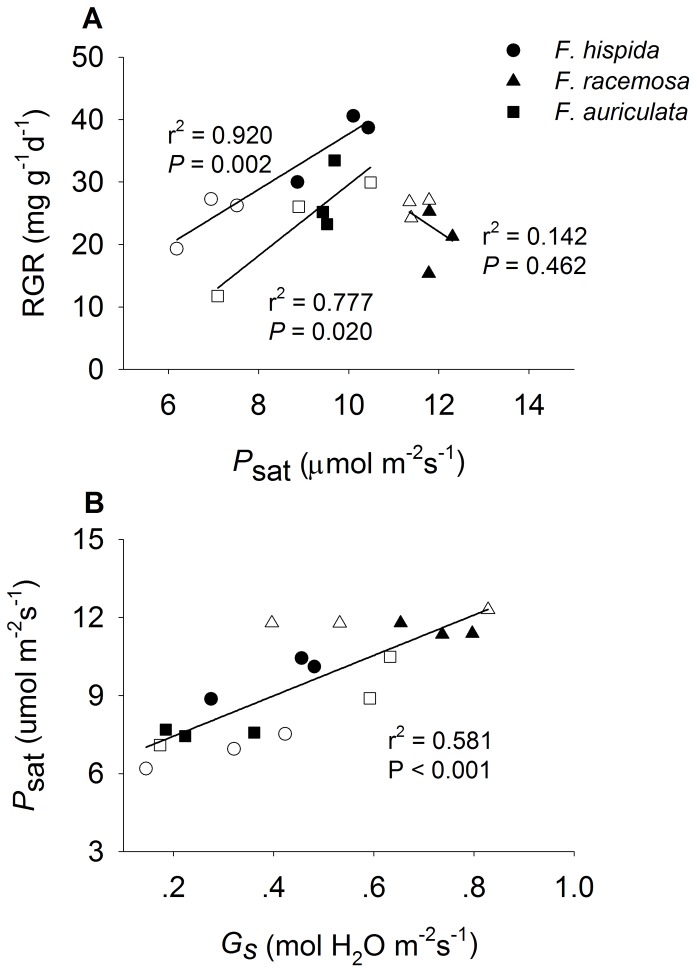
RGR as a function of *P*_sat_ and *P*_sat_ as a function of *G*_s_ in *Ficus* saplings under two soil nutrient levels. Significant differences between treatments are marked with different letters (mean ± SE, n = 3, *P*<0.05). Black shapes, fertile, white shapes, infertile. RGR relative growth rate (mg·g^−1^·d^−1^); *P*_sat_, light saturated photosynthetic rate (μmol m^−2^s^−1^); *G*_s_, stomatal conductance (mol H_2_O m^−2^s^−1^).

**Figure 5:**
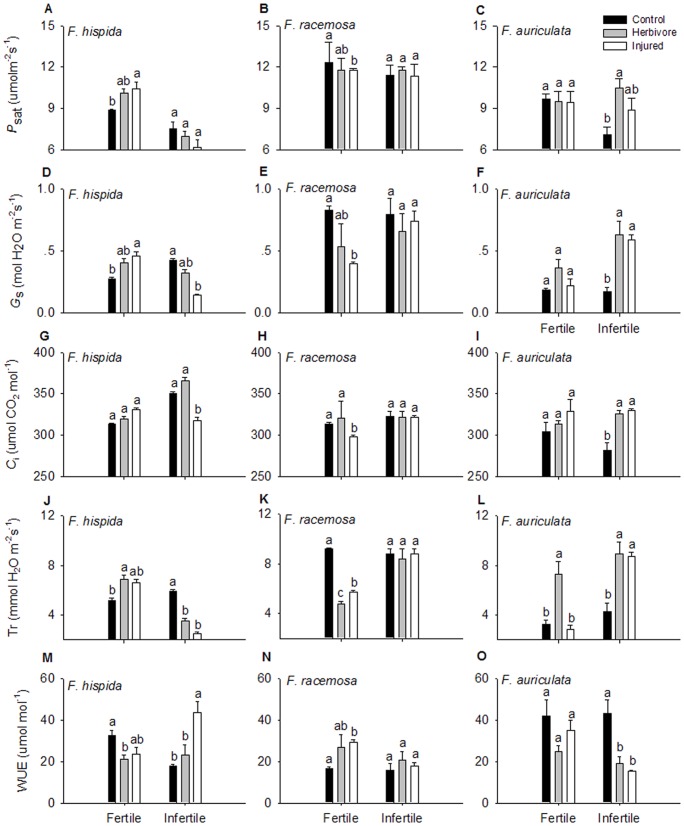
Effect of treatments on photosynthetic characteristics of *Ficus* saplings under two soil nutrient levels. Significant differences between treatments are marked with different letters (mean ± SE, n = 3, *P*<0.05). *P*_sat_, light saturated photosynthetic rate (μmol m^−2^s^−1^); *G*_s_, stomatal conductance (mol H_2_O m^−2^s^−1^); WUE, water use efficiency (μmol mol^−1^).

RGR increased linearly with increasing *P*_sat_ in *F. hispida* and *F. auriculata* (Fig. 4A). *P*_sat_ exhibited a positive relationship with *G*_s_ (Fig. 4B). A three-way ANOVA showed that *P*_sat_ was affected significantly by species (F = 41.77, *P*<0.001), nutrient level (F = 18.66, *P*<0.001), the interaction between species and nutrient level (F = 6.14, *P* = 0.005), and the interaction among species, nutrient level, and treatments (F = 2.60, *P* = 0.05). Herbivory and artificial injury increased *P*_sat_ in *F. hispida* saplings in fertile soils, but had no effect in infertile conditions (Fig. 5A). Both herbivory and artificial injury remarkably decreased the *P*_sat_ of *F. racemosa* under fertile conditions, but this was not the case under infertile conditions (Fig. 5B). For *F. auriculata*, *P*_sat_ increased prominently after herbivory and artificial injury under infertile conditions but showed no change under fertile conditions (Fig. 5C).

Species, soil nutrient, treatment and the interaction among three factors had significantly effect on *G_s_*, *C*_i_, Tr and WUE (*P*<0.05). The *G_s_*, *C*_i_ and Tr of *F. hispida* increased considerably after herbivore damage and artificial injury under fertile conditions but decreased dramatically under infertile conditions (Fig. 5D–L). Herbivore damage and artificial injury significantly inhibited *G_s_*, *C*_i_ and Tr in *F. racemosa* under fertile soil conditions but had no effect under infertile soil conditions. The *G_s_*, *C*_i_ and Tr of *F. auriculata* increased intensely after herbivore damage under both soil nutrient levels and increased significantly after artificial injury under infertile conditions. WUE of *F. hispida* decreased intensely following herbivore damage and artificial injury under fertile conditions, and increased under infertile conditions (Fig. 5M). The WUE of *F. racemosa* increased following herbivore damage and artificial injury under fertile conditions, but did not change significantly under infertile conditions (Fig. 5N). Herbivore damage and artificial injury caused the WUE of *F. auriculata* to decrease notably under both soil nutrient levels (Fig. 5O).

## Discussion

The three *Ficus* species showed great variation in soil nutrient associated compensatory regrowth. Overcompensatory regrowth in *F. hispida* was observed only under fertile soil conditions and *F. hispida* displayed full compensatory regrowth under fertile soil conditions. In *F. auriculata*, overcompensatory regrowth only occurred under infertile soil conditions, full compensatory regrowth were showed under fertile soil conditions. Meanwhile, *F. racemosa*, was less sensitive to the soil nutrient treatment and did not show significant overcompensatory regrowth under either soil nutrient condition. Both introduced insect herbivores and artificial damage had a significant effect on compensatory regrowth in any of the three *Ficus* species. This study provided experimental results for the conditionality of plant response to herbivore damage, and indicates that a general prediction on compensatory regrowth for all species is almost impossible.

### Interspecific differences in compensatory regrowth

The saplings of three *Ficus* species displayed interspecific variation in the degree of compensatory regrowth following herbivore damage and artificial injury under both fertile and infertile conditions. The significant overcompensatory regrowth of *F. hispida* occurred only in the high nutrient soil (Fig. 2). This pattern has been demonstrated in many other studies [12], [29]. However, based on a meta-analysis from Hawkes and Sullivan [41], compensatory regrowth in monocot plants is more common when resources are high, but not for dicot herbs and woody plants. In another review [9], out of the 48 cases examined, only 31% showed greater tolerance in high-resource conditions. In contrast to *F. hispida*, overcompensatory regrowth in *F. auriculata* saplings was observed under low rather than high nutrient conditions, which is supported by other studies [9], [34].

In this study, both *F. hispida* and *F. auriculata* showed a significant increase in biomass under fertile soil conditions compared to infertile soil conditions (Fig. 2), which is consistent with the finding that soil nutrient level is the limiting factor for these two species. On the other hand, *F. racemosa* did not show a significant change under different soil nutrient levels, which suggests that soil nutrient level is not a limiting factor for this species (Fig. 2). For *F. hispida*, soil nutrient level was the limiting factor, and herbivory on leaves decreased this limiting resource. Eventually, adding the resource (high soil nutrient level) ameliorated the impact of herbivory and increased tolerance. For *F. auriculata*, herbivore feeding might exacerbate carbon limitation, which is limiting under high nitrogen conditions. Therefore, tolerance of *F. auriculata* saplings in fertile soil was lower. In contrast, soil nutrient level was not a limiting factor for the saplings of *F. racemosa*. The consumed leaf area of herbivory insect might be insufficient to affect the utilization of soil nitrogen for *F. racemosa*, or it might only affect other local resources. Accordingly, *F. racemosa* showed equal tolerance at both high and low soil nutrient levels.

### Photosynthesis and compensatory regrowth

An increase in photosynthetic capacity following herbivore feeding and artificial injury appeared to be one of the mechanisms for biomass increment in this study. Both herbivore damage and artificial injury enhanced *P*_sat_, *G*_s_ and *C*_i_ in *F. hispida* (in fertile soil) and *F. auriculata* (in infertile soil), thus resulting in the observed increase in RGR (Fig. 1, 2). Herbivory-induced compensatory regrowth is a rather common phenomenon, although other research has indicated that insect herbivory may decrease the photosynthetic capacity in the remaining leaf tissue [42]–[45]. Compensatory regrowth may result from an increase in carboxylation efficiency or rate of transpiration [46], [47]. In this study, *C*_i_ of *F. hispida* and *F. auriculata* increased intensely after herbivory feeding and artificial damage which confirmed the increase in utilization of carbon dioxide. Additionally, WUE of *F. hispida* and *F. auriculata* decreased largely following treatments, indicating a trade-off between photosynthesis and the utilization of water [48], [49]. Herbivore damage and artificial injury increased the intercellular CO_2_ and the rate of transpiration which resulted in the increase of photosynthesis (Fig 4, 5). The sink demand within the leaf may also be affected by herbivore damage, which is well documented in the extensive literature that exists on photosynthetic compensatory regrowth in response to arthropod herbivory [6], [45]. Indirect alterations of photosynthesis have been identified across multiple plant systems and can be categorized by plant responses [45]. Precisely how the indirect effect of photosynthesis propagates away from the point of damage remains unknown. While for *F. racemosa*, the results showed that there was no relationship between photosynthesis and RGR and indicated that the full compensatory regrowth of this species might result from modification of other characteristics such as leaf area.

### Differences in biomass allocation

The differing biomass allocation of the saplings might be the other mechanism of compensatory regrowth in these three *Ficus* species. In the two species that showed significant overcompensatory regrowth, *F. hispida* and *F. auriculata*, the biomass allocation of the extra growth differed. In *F. hispida*, the enhancement in biomass occurred mostly above ground, while in *F. auriculata*, the enhancement occurred mostly below ground. Many other studies have also shown that the increase in biomass as a result of compensatory response was allocated to shoots [2], [20], [50]. In *Populus*, defoliation increased shoot biomass even at the expense of decreased root biomass [51].

The compensatory regrowth observed in *F. auriculata* is in contrast with the majority of published data on compensatory regrowth following herbivory [51]–[54]. The root/shoot ratio of *F. auriculata* increased in response to both herbivore damage and artificial injury, which indicates that this species allocated more resources to root biomass (Fig. 3). This overcompensatory regrowth occurred at the low nutrient level; therefore, an increase in biomass of the root system may help damaged plants better acquire nutrients for regrowth [5], [6], [11]. From an ecological viewpoint, a temporary storage of biomass in the roots could be beneficial as a type of herbivore defense.

### Herbivore damage vs. artificial injury

Our results showed that both herbivore damage and artificial injury stimulated similar responses in the three *Ficus* species under both fertile and infertile soil conditions. Previous studies have suggested that some plant species respond differently according to damage type. In some plants, defense genes may be responsive to insect feeding but not to physical damage alone [22], [23], and such responses can often be mimicked with insect-derived cues found in regurgitant [55]–[57]. For example, *Nicotiana sylvestris* (Solanaceae) concentrated more jasmonic acid (JA) following herbivore feeding than mechanical damage [24],while some studies have shown that mechanical wounding and herbivore damage induce similar responses [58]. In our study, the three *Ficus* saplings responded to herbivore damage and aritificial injury in the same way, indicating that *Ficus* saplings are sensitive to the loss of leaf area. The results also suggest that species may have responded similarly because the insect herbivore is not yet specialized or the plant species have not adapted locally to recognize the presence of this insect damage which has been recently demonstrated that even within a plant-herbivore system, a specific plant population can differ in the ability to recognize its local versus foreign herbivore [59].

## Conclusion

This study demonstrated that the compensatory regrowth, as a response to herbivory in saplings, and the effect of nutrient levels on such regrowth, varied across different species of the same genus, which is consistent with other studies [60]–[62]. The increase of photosynthesis and differences in biomass allocation appear to be the mechanisms underlying compensatory regrowth in *Ficus* saplings. The different strategies for three *Ficus* species sapling were consistent in both glasshouse and field experiment, which suggest the mechanism may operate in nature environments. However, our study did not manipulate the effect of water resources, which might limit the WUE. Future studies should consider other potential limiting factors in trying to elucidate the effect of resource availability on the response of plants following damage. *Ficus* species displayed different degrees of compensatory regrowth at the 20^th^ d following damage treatments, and longer-term experiments should be considered to determine whether the degree of compensation varies with time after damage. Tropical rainforests are the most complex territorial ecosystems on the planet, and to explore the mechanisms for the maintenance of complexity and species coexistence in the tropical rainforest is one of the fundamental questions for ecology [12], [63]. Future research is needed to explore how saplings utilize different defensive strategies to survive and maximize recruitment in nature and to determine the change in such strategies for different plant species throughout their ontogeny [64].

## Materials and Methods

### Study site, soil treatment and sapling preparation

All experiments were conducted at the Xishuangbanna Tropical Botanical Garden (XTBG), Chinese Academy of Sciences (21°41′N, 101°25′E; 570 m asl; annual mean temperature, 21.5°C; annual mean rainfall, 1560 mm). The study included two experiments: the first was done in an insect-proof glasshouse and the second was performed in the field. For the glasshouse experiment, all the saplings was placed in an insect-proof and rain-proof glass house. One white and two black nylon shade networks created 10% irradiance, similar to levels of irradiance in field conditions. For the experiment in the field, saplings were placed in a secondary natural forest with canopy coverage being approximately 80–90%.

Two levels of soil nutrient were set up for the experiments. For the low-level nutrient treatment, soil was collected from above the deep soil layer with a total N content of 0.99 g/kg, and for the high-level nutrient treatment, fertilizers (N-P-K  =  15-15-15) were added to the low-level soil, yielding a total N content of 1.86 g/kg. The total N content was determined by Dumas combustion analysis [65] using an elemental analyser (Vario MAX CN, Germany) by the Biogeochemical Laboratory of the Kunming Division of the XTBG. All the experiments was conducted in pots (diameter, 20 cm) with one sapling per pot.

Seeds of the three *Ficus* species were collected from approximately 15–30 ripe fruits on 3–5 individual trees (5 trees for *F. hispida* and *F. racemosa*, 3 trees for *F. auriculata*) in the nearby forest. Seeds were germinated in washed sand. After 8 weeks, seedlings of approximately the same size (30∼50 cm) from each species were transplanted into the pots and assigned randomly to one of two groups for the soil nutrient treatment.

### Herbivore treatment and biomass measurement

About twelve weeks after transplant, five saplings in each soil nutrient condition (i.e., 10 saplings per species) were used to determine the initial biomass (measured separately by shoot and root biomass). The remaining saplings of each species in each soil nutrient condition were used for herbivore compensatory-regrowth experiments.

Third instar caterpillars of a common lepidopteran herbivore of *Ficus* (*Asota caricae* Fabricius) were prepared for the experiment in the glasshouse. The larvae were deprived of food for 24 h preceding the experiment to ensure that damage treatments could be completed within a single day.

The glasshouse experiment was conducted in August, 2009. Leaf damage was simulated for each *Ficus* species in both soil conditions using 5 duplicates as follows: A) Control: Saplings without damage by larvae or hole punch; B) Herbivore damage: Five prepared larvae were placed on the youngest mature leaf for 24 h and covered with insect-proof net; C) Artificial injury by hole punch (5 mm diameter): The first mature leaf was punched six times (we injured the youngest leaf once every 4 h and ensured that the size and shape of the removed area matched that in the herbivore damage treatment). Damage to the first mature leaf was about 30% in both herbivore treatments and artificial injured saplings, equal to about 7% of total leaf mass (more larvae were added if the removed leaf area was less than 30%).

The field experiment was conducted in May 2011. Saplings of each of the three *Ficus* species were planted in both high and low nutrient soils (as described above), and exposed to two treatments: A) Contol: herbivore-free, and, B) exposure to natural herbivory. All the saplings of *Ficus* species were departed into four groups. Each of the four groups included 6 saplings of each species in each soil nutrient condition, within a group, placed >1 m apart, and at >30 m distances between groups.

All saplings were harvested 20 days after treatment both in the glasshouse and in the field. Leaf area consumed by natural herbivore (control) were measured by a LI-3000 portable area meter (Li-Cor, Lincoln, USA). Soil was cleaned from the roots. The saplings were oven-dried at 40°C until constant masses were reached and then were separated into shoot and root. Total biomass was measured and the relative growth rate (RGR) was determined by the following equation [66]:

RGR (mg·g^−1^·d^−1^)  =  [ln(sapling mass at harvest)–ln(initial sapling mass)]/[duration of study (d)].

The degree of compensatory regrowth was determined by comparing the total biomass of damaged and undamaged saplings. If the biomasses of damaged and undamaged saplings are similar, we can infer full compensatory regrowth of damaged saplings, higher indicates overcompensatory regrowth and less indicates under compensatory regrowth.

### Photosynthesis measurement

Photosynthetic characteristics were measured only in the glasshouse experiment using two Li-6400 portable photosynthesis systems (Li-Cor, Lincoln, Nebraska, USA) (n = 3). Three untreated leaves were measured on each sapling. The measurements were taken 20 days after the herbivore treatment, between 0800 and 1400 on 15 August. In the leaf chamber, each leaf was acclimated to 500 µmol·m^−2^·s^−1^ Photosynthetic Photon Flux Density (PPFD) for 1∼3 min and then to 1000 µmol·m^−2^·s^−1^ for 1∼3 min prior to gas exchange measurements. This method was found to induce plant photosynthesis without causing photoinhibition. Light saturated photosynthetic rates (*P*_sat_), stomatal conductance (*G_s_*), internal leaf CO_2_ concentration (*C*_i_), and transpiration rate (Tr) were recorded when the sample leaf was balanced for 200 s under saturated PPFD. Water use efficiency (WUE) was calculated as the ratio of *P*_sat_ to *G*_s_. During photosynthesis measurements, cuvette air was maintained at 27∼29°C, 70∼80% relative humidity, and 360 µmol·mol^−1^ sample CO_2_ partial pressure.

### Statistical analysis

Three-way ANOVA was performed to test the effects of species, soil nutrient level, treatments, and all possible interactions between these factors on growth, biomass allocation and photosynthetic characters (The data of RGR, root/shoot ratio, *P*_max_, *C*_i_, Tr and WUE were normally distributed and homogenous. Data of *G*_s_ were log10-transformed which made the residuals reasonably normal and homogenous. And the data of one sapling of *F. racemosa* which was totally consumed in field were deleted to avoid bias of results.). Because the effect of interactions between these factors on these variables were significant, one-way analysis and multi-comparison (Tukey-HSD) of variance was performed to test treatment effects on RGR, root/shoot ratio and photosynthetic characters for each species under each soil nutrient level. To determine whether soil nutrient level was the limiting resource for all three *Ficus* saplings, *t*-tests were performed on the total mass of the undamaged saplings of each species. One-way ANCOVA was used to test differences among the three *Ficus* species in the correlation between *P*_sat_ and RGR, *P*_sat_ and *G*_s_, with species as a fixed factor, and variables indicated by y- and x-axes as dependent variable and covariate, respectively. If the difference was significant, we then tested for the significance of the correlation (Pearson correlation, two-tailed) for the three *Ficus* species separately; otherwise, we pooled data from all species to test for the significance of correlation. To evaluate the effects of species, soil nutrient level and their interactions on consumed leaf area in the field experiment, two-way ANOVA and multi-comparison (Tukey-HSD) were performed, where the average consumed leaf area of each species with each soil nutrient level for each group were analyzed (n = 4). The data were normally distributed and homogenous. All statistical analyses were performed using SPSS (SPSS 13.0, Chicago, USA).
